# Empty-nest-related psychological distress is associated with progression of brain white matter lesions and cognitive impairment in the elderly

**DOI:** 10.1038/srep43816

**Published:** 2017-03-03

**Authors:** Dandan Duan, Yuanli Dong, Hua Zhang, Yingxin Zhao, Yutao Diao, Yi Cui, Juan Wang, Qiang Chai, Zhendong Liu

**Affiliations:** 1 School of Medicine and Life Sciences, University of Jinan-Shandong Academy of Medical Sciences, Zhangqiu, Shandong 250200, China; 2 Cardio-Cerebrovascular Control and Research Center, Institute of Basic Medicine, Shandong Academy of Medical Sciences, Jinan, Shandong, 250062, China; 3 Department of Community Service, Lanshan District People’s Hospital, Linyi, Shandong, 276002, China; 4 Department of Radiology, Qilu Hospital of Shandong University, Jinan, Shandong, 250012, China; 5 Department of Cardiology, The Second Hospital of Shandong University, Jinan, Shandong, 250000, China

## Abstract

This study evaluated the association between empty-nest-related psychological distress and the progression of white matter lesions (WMLs) and cognitive impairment in 219 elderly subjects aged 60 years or over. Psychological distress was assessed using the University of California at Los Angeles Loneliness Scale (UCLA-LS) and Geriatric Depression Scale (GDS) Short-Form. Cognitive function was evaluated using the MMSE and MoCA. White matter hyperintensities (WMH) were assessed using magnetic resonance imaging. After 5.2-year follow-up, the reductions in MMSE and MoCA scores and the increases in periventricular (P)WMH, deep (D)WMH, and total WMH volumes in the empty-nest elderly were greater than those in the non-empty-nest elderly (*P* < 0.05). The reduced MMSE and MoCA scores and increased volumes of PWMH and total WMH in the empty-nest elderly living alone were greater than those in the empty-nest elderly living with a spouse (*P* < 0.05). UCLA-LS and GDS scores were significantly and independently associated with reduced MMSE and MoCA scores and the increased volumes of PWMH, DWMH, and total WMH. The results indicate that empty-nest-related psychological distress is associated with progression of WMLs and cognitive impairment in the elderly.

Empty-nest elderly are those who do not live with their children^1^,^2^. They live either with a spouse (empty-nest couple) or alone (empty-nest single)^2^,^3^. Along with extended average life expectancy, increasing immigration, and rapid economic development, the number of empty-nest elderly is gradually increasing in both developed and developing countries^4^,^5^. In the United States, the percentage of empty-nest elderly males aged 60 years or over rose from 19 to 78% between 1880 and 2000^4^. In China, the number of empty-nest elderly was 100 million, accounting for approximately 50% of the total elderly population in 2013^2^. Making matters worse, it is projected that the proportion will reach 90% in 2030^2^. Furthermore, recent research has shown that the empty-nest elderly are at a higher risk of experiencing catastrophic health expenditures than the non-empty-nest elderly^6^. We believe that, in the near future, the empty-nest elderly will become an unavoidable social issue in the aging process worldwide.

Epidemiological studies have indicated that the empty-nest elderly commonly suffer from loneliness, depressive symptoms, and mental health problems^1^,^7^,^8^. It is reported that approximately 43.6% of the empty-nest elderly experience moderate loneliness and 10.9% suffer serious loneliness in rural areas of China^8^. In European cities, the prevalence of depression is approximately 12.3% in the community-dwelling population above 65 years of age^9^.

Loneliness and depressive symptoms are deemed unpleasant state experiences and serious problems among the elderly^1^,^7^,^8^. There is a mutually promoting function between loneliness and depressive symptoms^10^. They exhibit deleterious effects on mental wellbeing as well as on physical health. Evidence indicates that both loneliness and depressive symptoms are significant risk factors for hypertension^11^, vascular stiffness^12^,^13^, metabolic syndrome^14^, and dementia^10^,^15^.

Cognitive impairment is a common chronic condition with aging and interferes with daily functioning and wellbeing^16^. It is estimated that the prevalence of cognitive impairment is 17 to 34% in the general older population aged 60 years or over^17^,^18^. The burden of caring for those with cognitive impairment is immense^19^. It is thus necessary to understand the risk factors of cognitive impairment to develop preventive and therapeutic measures.

White matter comprises over half of the human brain and is regarded as the substrate for cognition. It is the essential connection of distributed neural networks. White matter hyperintensities (WMH), a subtype of small vessel disease, are an important indicator of white matter lesions (WMLs) and are associated with an increased risk of cognitive impairment and dementia^20^,^21^,^22^,^23^. WMH are frequently detected in structural brain scans using magnetic resonance imaging (MRI).

Few studies have been performed to investigate how loneliness correlates with cognitive impairment^24^ and WMH^25^,^26^,^27^. O’Luanaigh and colleagues^24^ reported that loneliness was independently associated with impaired global cognition after adjusting for social networks and depression. Johansson and coworkers^25^ found that long-standing psychological distress in midlife increases the risk of WMLs on computed tomography in late life. In young adults, brain white matter structure changes were found to be related to loneliness^28^.

However, the association of empty-nest-related psychological distress with progression of cognitive impairment and WMLs has not been explored in depth in the elderly. We performed a 5-year follow-up study to investigate and elucidate the association between empty-nest-related psychological distress and the progression of WMLs and cognitive impairment in the elderly.

## Results

### Study participants and baseline demographic and clinical characteristics

**Figure 1:**
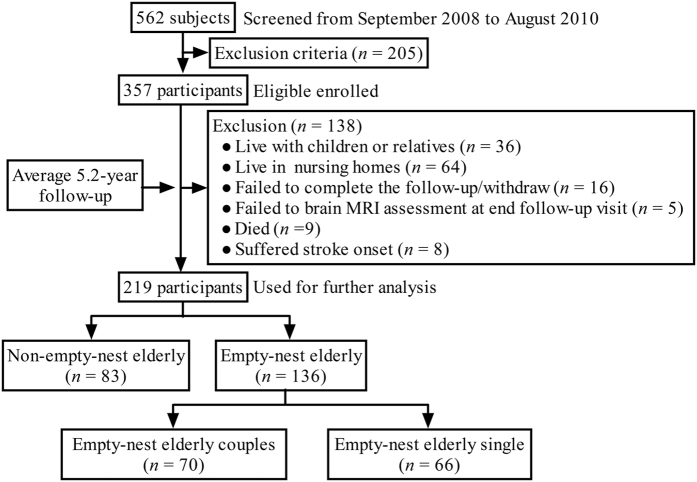
Flowchart of subject enrollment and screening.

A flow diagram of the study is presented in Fig. 1. Among 357 participants, 138 were excluded for the following reasons: 36 lived with children or relatives, 64 lived in nursing homes, 16 failed to complete the follow-up or had withdrawn, 9 died, and 8 suffered from the onset of stroke. Finally, 219 participants completed the study and were used for further analysis. Among them, 83 were non-empty-nest elderly (control group), 70 were empty-nest elderly living with a spouse (couples group), and 66 were empty-nest elderly living alone (single group). Table 1 summarizes the baseline demographic and clinical characteristics of participants in the three groups.

### Baseline variables of psychological outcomes, global cognitive function, and brain MRI

Table 2 reveals the baseline variables of psychological outcomes, global cognitive function, and WMLs in the three groups. The University of California at Los Angeles Loneliness Scale (UCLA-LS) and Geriatric Depression Scale (GDS) scores increased from controls to couples to singles. Differences in the UCLA-LS and GDS scores were significant in every pairwise comparison between groups (all *P* < 0.001). There were no significant differences in the variables of global cognitive function and brain MRI among the three groups.

### Changes in global cognitive function among the three groups

**Figure 2:**
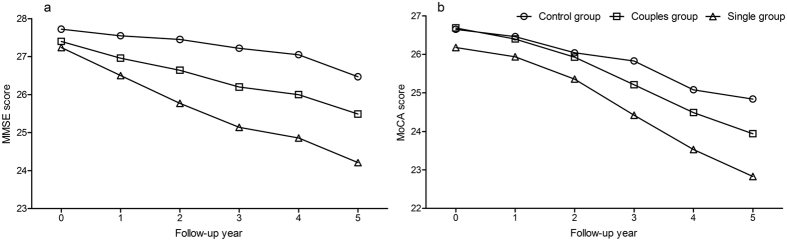
Trends of global cognitive impairment in the elderly in relation to different lifestyles at the annual measurement over a 5-year follow-up period. Results are presented as the mean. (**a**) Trends of the mean MMSE scores at the annual measurement in three groups; (**b**) trends of the mean MoCA scores at the annual measurement in three groups. MMSE, Mini-Mental State Examination; MoCA, Montreal Cognitive Assessment.

Decreasing trends were found in the mean Mini-Mental State Examination (MMSE) and Montreal Cognitive Assessment (MoCA) scores in the three groups during the study (Fig. 2). The results of repeated measures analysis of variance showed that there were significant differences in the mean MMSE and MoCA scores between the three groups at the annual measurement (for MMSE scores, *F* value = 15.793, *P* < 0.001; for MoCA scores, *F* value = 9.119, *P* < 0.001). Differences in the mean MMSE scores were significant in every pairwise comparison between groups (all *P* < 0.05). Differences in the mean MoCA scores between the single group and the other two groups were significant (both comparisons *P* < 0.05).

**Figure 3:**
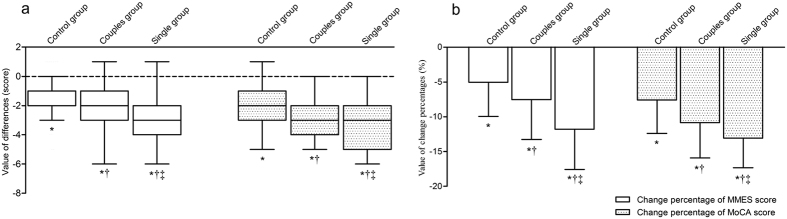
Progression of global cognitive impairment in the elderly in relation to different lifestyles over a 5-year follow-up period. Results are presented as the mean ± standard deviation or median with IQR. (**a**) Differences in the score of MMSE and MoCA between baseline and final follow-up among three groups; (**b**) changes in the percentages of score of MMSE and MoCA between baseline and final follow-up among three groups. MMSE, Mini-Mental State Examination; MoCA, Montreal Cognitive Assessment. *P < 0.05, compared to baseline; ^†^*P* < 0.05, compared to control group; ^‡^*P* < 0.05, compared to couples group.

Figure 3 demonstrates the changes in MMSE and MoCA scores over the course of the follow-up in the three groups. The MMSE and MoCA scores were significantly reduced in all three groups (*P* < 0.05). The reduction in MMSE and MoCA scores increased from controls to couples to singles. Differences in the reduction of MMSE and MoCA scores were significant in every pairwise comparison between groups (all *P* < 0.05). After adjustments were made for baseline values, the percentage reduction of MMSE and MoCA score still increased from controls to couples to singles. Differences in the percentage reduction of MMSE and MoCA scores were significant in every pairwise comparison between groups (all *P* < 0.05).

### Changes in brain WMH among the three groups

**Figure 4:**
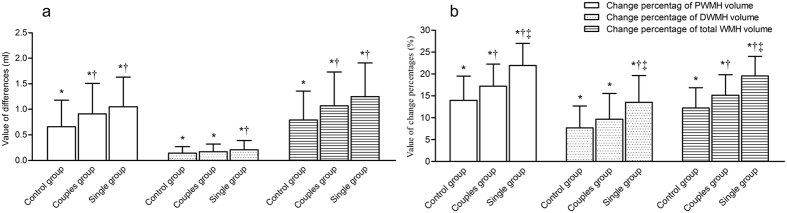
Progression of white matter lesions in the elderly in relation to different lifestyle over a 5-year follow-up period. The results are presented as the mean ± standards. (**a**) Differences in the volume of PWMH, DWMH, and total WMH between baseline and final follow-up among three groups; (**b**) change percentages of volume of PWMH, DWMH, and total WMH between baseline and final follow-up among three groups. PWMH, periventricular white matter hyperintensities; DWMH, deep white matter hyperintensities; WMH, white matter hyperintensities. *P < 0.05, compared to baseline; ^†^*P* < 0.05, compared to control group; ^‡^*P* < 0.05, compared to couples group.

The volume of PWMH, DWMH, and total WMH had increased markedly by the end of the trial compared with baseline in all three groups (all *P* < 0.001) (Fig. 4). The increase in volume of PWMH and total WMH in the couples group and the single group were greater than those in the control group (*P* < 0.05). The increase in volume of DWMH in the single group was greater than that in the control group (*P* < 0.05).

Meanwhile, we compared the percentage increases in volume of PWMH, DWMH, and total WMH, which were adjusted for baseline values, among the three groups. The percentage increases in volume of PWMH and total WMH increased from controls to couples to singles. Differences in the percentage increases in volume of PWMH and total WMH were significant between any two groups (all *P* < 0.001). The percentage increase in volume of DWMH in the single group was statistically higher than those in the control and couples groups (*P* < 0.001).

### Correlation of psychological outcome with global cognitive function and brain WMH in all participants

Table 3 reveals the correlations of UCLA-LS and GDS scores with the changes in MMSE, MoCA, PWMH, DWMH, and total WMH during the follow-up period in all participants. The correlations of UCLA-LS and GDS scores with the differences in MMSE and MoCA scores were assessed using Spearman correlation analysis due to the non-normality of differences in the MMSE and MoCA scores. The UCLA-LS and GDS scores were significantly and negatively correlated with the changes in MMSE and MoCA scores, even after they were adjusted for baseline values (all *P* < 0.001). In contrast, the UCLA-LS and GDS scores were significantly and positively correlated with changes in PWMH, DWMH, and total WMH (*P* < 0.001, respectively). Furthermore, the strength of the correlations became greater after the volumes of PWMH, DWMH, and total WMH were adjusted for their respective baseline values (all *P* < 0.001).

### Multiple linear backward stepwise regression analysis

Multiple linear backward stepwise regression analysis was performed to explore factors that were independently associated with the changes in global cognitive function and brain WMH (Table 4). The changes in MMSE, MoCA, PWMH, DWMH, and total WMH were used as dependent variables. Independent variables included UCLA-LS, GDS, age, sex, smoking, alcohol consumption, education, body mass index, history of hypertension, use of antihypertensive agents, history of diabetes mellitus, use of hypoglycemic agents, blood pressure, and fasting blood lipid and glucose levels. Independent variables also included baseline total WMH in the models of changes in MMSE and MoCA. Baseline MMSE score was also included as an independent variable in the models of changes in total WMH, PWMH, and DWMH.

UCLA-LS score, GDS score, education, and low-density lipoprotein cholesterol were significantly correlated with reduced MMSE scores (adjusted R square = 0.344, all *P* < 0.05). UCLA-LS score, GDS score, education, and antihypertension were significantly correlated with reduced MoCA scores (adjusted R square = 0.363, all *P* < 0.05). UCLA-LS score, GDS score, systolic blood pressure, hypertension history, and antihypertension were significantly correlated with increased volume of PWMH (adjusted R square = 0.113, all *P* < 0.05). UCLA-LS score, GDS score, total cholesterol, fasting plasma glucose, body mass index, and antihypertension were significantly correlated with increased volume of DWMH (adjusted R square = 0.107, all *P* < 0.05). UCLA-LS score, GDS score, fasting plasma glucose, hypertension history, and antihypertension were significantly correlated with increased volume of total WMH (adjusted R square = 0.135, all *P* < 0.05). Details of the analysis results are summarized in Supplement Table 1a.

For percentage reduction of MMSE score, the significantly correlated variables were UCLA-LS score, GDS score, education, and low-density lipoprotein cholesterol (adjusted R square = 0.361, all *P* < 0.05). For percentage reduction of MoCA score, the remarkable results were UCLA-LS score, GDS score, education, and antihypertension (adjusted R square = 0.378, all *P* < 0.05). For percentage increase in the volume of PWMH, the significant results were UCLA-LS score, GDS score, and antihypertension (adjusted R square = 0.382, all *P* < 0.05). For percentage increase in the volume of DWMH, the significant results were UCLA-LS score, GDS score, body mass index, and fasting plasma glucose (adjusted R square = 0.261, all *P* < 0.05). For percentage increase in the volume of total WMH, the significant results were UCLA-LS score, GDS score, and antihypertension (adjusted R square = 0.406, all *P* < 0.05). Details of the analysis results are summarized in Supplement Table 1b.

Most importantly, the results of multiple linear backward stepwise regression analysis showed that UCLA-LS score and GDS score were always significantly and independently associated with changes in MMSE, MoCA, PWMH, DWMH, and total WMH.

## Discussion

The primary objective of the current study was to determine whether there were independent contributions of loneliness and depressive symptoms to the progression of brain WMLs and cognitive impairment in the empty-nest elderly. The major findings were as follows: (1) MMSE and MoCA scores were significantly reduced, and the volumes of PWMH, DWMH, and total WMH were significantly increased in all participants during the follow-up period. (2) The empty-nest elderly, especially the empty-nest elderly living alone, suffered more serious progression of brain WMLs and cognitive impairment than the non-empty-nest elderly. (3) The empty-nest-related loneliness and depressive symptoms assessed using UCLA-LS and GDS significantly and independently contributed to the progression of WMLs and cognitive impairment in the elderly.

Empty-nest elderly were more prone to loneliness and depression than non-empty-nest elderly^8^. Our results agreed with previous studies^1^,^7^,^8^. Loneliness and depressive symptoms in empty-nest elderly were more serious than those in non-empty-nest elderly. Empty-nest elderly living alone suffered more serious loneliness and depressive symptoms than empty-nest elderly living with a spouse. The reason may be that the empty-nest elderly, especially the empty-nest elderly living alone, suffer from poor social relationships and more chronic psychological distress^1^,^7^,^8^.

Studies^29^,^30^,^31^,^32^ have demonstrated that cognitive impairment is closely associated with social relationships. A systematic review and meta-analysis study^29^ showed that less frequent social contacts and more loneliness are markedly associated with the incidence of cognitive impairment and dementia. However, good social relationships can delay or prevent the onset of multiple adverse psychological conditions, such as loneliness^1^, depression^30^, and memory complaints^31^,^32^.

In the present study, cognitive function in the empty-nest elderly was significantly decreased compared with the non-empty-nest elderly during the 5-year follow-up period. Moreover, the cognitive function of the empty-nest elderly living alone declined more steeply than that of the empty-nest elderly living with a spouse. The results of correlation and regression analysis showed that the scores of UCLA-LS and GDS were independently and positively correlated with reduced MMSE and MoCA scores. The correlations were retained even after adjusting for covariates and baseline MMSE and MoCA scores.

Changes in brain structure induced by loneliness and depressive symptoms may be one potential explanation for why more loneliness and depressive symptoms exacerbated the progression of cognitive impairment. Nakagawa and coworkers^28^ found that white matter structures relate to loneliness in young adults. Stern and colleagues^33^ reported that brain structure can be affected by social ties and results in more efficient use of cerebral networks. Neurogenesis^33^,^34^ and synaptic density^34^,^35^ have been identified to be closely related to an engaged lifestyle and stimulating environments. Psychological distress is correlated with neurobiological mechanisms other than pathological hallmarks such as tangles and cortical plaques^29^,^36^.

In our study, PWMH and total WMH in the empty-nest elderly became more serious than those in the non-empty-nest elderly over the duration of follow-up. The burden of PWMH, DWMH, and total WMH in the empty-nest elderly living alone grew more serious than those in the empty-nest elderly living with a spouse after adjustment for the baseline burden of PWMH, DWMH, and total WMH. In agreement with the progression of cognitive impairment, UCLA-LS and GDS scores were independently and positively correlated with progression of WMLs, even after adjustment for covariates. This reveals that empty-nest-related unpleasant states such as loneliness and depressive symptoms may play a crucial role in exacerbating the burden of WMH as well as the progression of cognitive impairment in the elderly.

There are some arguments about the clinical significance of WMH localized in different regions and their distribution in the brain^37^,^38^. Although there is not a uniformly accepted classification system, WMH consists of at least two subcategories, namely, PWMH and DWMH, which may have different but overlapping pathogenetic mechanisms^39^,^40^. PWMH is age-related and associated with ventricular enlargement. DWMH is pathologically heterogeneous and consists of focally widened spaces lined with atrophic myelin centered on fibrohyalinized vessels^41^. Moreover, WMH is subject to a strong genetic influence, but it is not uniform through the brain^40^. Sachdev^40^ reported that genetic influence is higher for DWMH than PWMH. We classified total WMH into PWMH and DWMH to explore the relationship of empty-nest and related psychological distress with the progression of WMLs. The results demonstrated that living arrangement is an important factor for the progression of PWMH as well as DWMH. In addition, loneliness and depressive symptoms were found to be independently associated with the progression of PWMH and DWMH, although there were differences in the strength of these associations.

A major strength of the present study is that it is a longitudinal and observational study. Participants underwent follow-up for 5.2 years on average. Another is strength that the empty-nest elderly group was further subdivided into those living with a spouse and those living alone. Those living alone were suffering more serious loneliness and depressive symptoms than those living with a spouse. Severe loneliness and depressive symptoms led to an accelerated progression of cognitive impairment and WMLs. In addition, those of the elderly who had never had children were excluded from the present study.

Although our study has a number of strengths, several limitations must be considered. First, elderly with hypertension and/or diabetes were included in the present study. Hypertension and diabetes are known to be important risk factors for WMLs and cognitive impairment. This may have induced a potential bias in our results, although we adjusted for history of hypertension and diabetes as well as antihypertension and anti-diabetes treatments. Second, 138 participants were excluded for various reasons during the follow-up period. The elimination rate was 37.91%, which might have influenced the results of our study. Third, there were significant differences in the education level between the control and the single group. Education is a powerful factor for effects of age on cognitive function^42^. Fourth, the sample size of our study was small. Large-scale and multicenter longitudinal studies are needed in the future. In addition, the incidence of dementia was not analyzed in the present study.

## Conclusions

Empty-nest-related psychological distress is associated with progression of brain WMLs and cognitive impairment in the elderly. Ensuring good social ties and minimizing psychological distress may be helpful in delaying or preventing the progression of WMLs and cognitive impairment in the elderly, especially in the empty-nest elderly living alone.

## Methods

### Study population and design

The present study was a longitudinal and observational study. From September 2008 to August 2010, 562 elderly people aged 60 years or over were screened from community dwellings in the area of Shandong, China. Among them, 357 participants found eligible were enrolled in the study. In the present study, empty-nest elderly were defined as those who have children but do not live with their children. Non-empty nest elderly were defined as those who live with their children. Exclusion criteria were as follows: childless elderly, dementia, Parkinson disease, schizophrenia, seizures, claustrophobia, bipolar disorder, brain tumor, secondary hypertension, end-stage heart disease, renal failure and dialysis treatment, connective tissue diseases, malignancy, contraindication to MRI, and unwillingness or difficulty in providing informed consent.

After the baseline survey, a clinical visit was conducted every 3 months for all participants. Demographic and clinical characteristics of participants were required at every clinical visit. Loneliness and depressive symptoms were assessed at baseline using the Chinese version of the UCLA-LS and GDS Short-Form, respectively. Global cognitive function was evaluated annually using the Chinese version of the MMSE and MoCA. WMLs were assessed at baseline and at the end of the trial using MRI. For participants with hypertension or diabetes mellitus, antihypertensive or hypoglycemic therapy had been advised by a cardiologist or endocrinologist if the participant was willing to receive treatment. As the present study had an observational design, there was no unified antihypertensive or hypoglycemic schedule for participants with hypertension or diabetes mellitus.

Written informed consent was obtained from all participants or their close relatives, if the participant was unable to write, in accordance with the ethical principles set forth in the Declaration of Helsinki. This study was approved by the Research Ethics Committee of the Shandong Academy of Medical Sciences.

### Measurement of psychological outcomes

Measurement of psychological distress was conducted using the Chinese version of the UCLA-LS^8^ and GDS Short-Form^27^,^43^ by a psychiatrist, who was blinded to the clinical and laboratory data, cognitive function, and brain MRI. UCLA-LS is a highly reliable and widely used questionnaire on social isolation loneliness^28^. It contains 20 items. There are 20 four-point Likert scale items on the UCLA-LS. Participants were asked to rate each item from 1 (Never) to 4 (Often) to assess how often they agreed with the description. Total scores on the UCLA-LS ranged from 20 to 80. A higher score reflects more loneliness experienced by that participant. The GDS, containing 15 one-point items, according to Yesavage, is an instrument with good internal consistency (alpha score = 0.85) for depression evaluation^44^,^45^. Each item has two possible answers: 1 (Yes) or 0 (No). Total GDS scores ranged from 0 to 15. Higher scores indicate more severe depression.

### Assessment of global cognitive function

Global cognitive function was assessed using the Chinese version of the MMSE^21^ and MoCA^46^ by a neuropsychology research assistant, who was an expert in cognitive function measurements. The assistant was blinded to the clinical and laboratory data, psychological outcomes, and brain MRI results. The MMSE is a validated, reliable, and powerful instrument for diagnosing global cognitive impairment^47^. It consists of five areas of possible cognitive impairment: orientation, registration, attention, calculation, and language. The MoCA has excellent test-retest and inter-rater reliability, and the internal consistency of the items was 0.884^48^. It includes nine areas of possible cognitive impairment: attention, concentration, executive functions, memory, language, visual-spatial ability, abstract thinking, calculation, and orientation. The maximal scores on the MMSE and MoCA are both 30 points. A lower score reflects more severe global cognitive impairment.

### Brain MRI protocol and processing

WMH was evaluated with MRI using a 3T GE Signa Horizon scanner (General Electric Medical Systems, Milwaukee, Wisc., USA) by a radiology expert according to the same protocol. The MRI sequences were as previously described^21^,^22^,^23^. Briefly, the T1-weighted 3D magnetization-prepared rapid gradient-echo sequence was isotropic 1 mm voxel, TR/TE/TI = 1900/3/900 ms, flip angle 9°; acquisition matrix 256 × 256 with 160 slices yielding 1 mm^3^ isotropic voxels; FOV 256 × 240 mm^2^, 1 mm thick slices, and no gap. The T2-weighted 3D fast spin-echo sequence was TR/TE = 3000/98 ms, FOV = 24 cm, acquisition matrix = 256 × 256, NEX = 0.5, 3 mm slice thickness, and no gap. FLAIR was TR/TE/TI = 5000/355/1800 ms, flip angle 120°, acquisition matrix 256 × 256 with 60 slices, 2 mm thick slices, and no slice gap.

WMH can be localized to the area immediately adjacent to the ventricles (periventricular WMH, PWMH) and the area under the cortex (deep WMH, DWMH). The total WMH volume was computed from an automated PWMH and DWMH segmentation on FLAIR axial images using Freesurfer^49^. The total WMH volume was calculated as the sum of PWMH and DWMH volume. All MRI analyses were conducted by a neuroradiologist, who was blinded to the lifestyle, psychological outcome, and cognitive function data of subjects.

### Covariates

Covariates included were, sex, age, smoking, alcohol consumption, education, body mass index, history of hypertension, use of antihypertensive agents, history of diabetes mellitus, use of hypoglycemic agents, blood pressure, and fasting blood lipid and glucose levels.

### Statistical analysis

Categorical data were presented as numbers with percentages. Numerical data were expressed in the form of mean ± standard deviation (SD) or median with interquartile range (IQR, the range from the 25^th^ to 75^th^ percentile) according to the normality of the data. The Kolmogorov-Smirnov test was used to assess the normality of data. A paired *t*-test or Mann-Whitney test was performed to assess the difference in variables between baseline and the final follow-up, depending on the normality of the data. Repeated measures analysis of variance with a Bonferroni post hoc test was applied to assess the differences in the primary outcome of MMSE and MoCA scores at annual measurement between three groups. The Greenhouse-Geisser test was used if Mauchly’s test of sphericity did not indicate data homogeneity. Change percentage was used to express the change of measured variables between baseline and the final follow-up. Change percentage was calculated as [(value at final follow-up − value at baseline)/value at baseline] × 100%. One-way analysis of variance (ANOVA) or the Kruskal-Wallis test was applied to detect the differences in numerical variables among groups according to the normality of data. The Bonferroni procedure with type I error adjustment or the Wilcoxon rank sum test was used to detect the difference between any two groups when a significant difference was found across all groups. The chi-square test was used to detect the differences in categorical data. The Pearson or Spearman correlation coefficient was determined to assess the relationship between two numerical parameters. Multiple linear backward stepwise regression analysis was used to explore whether any factors were independently associated with progression of WMH and cognitive impairment. The cut-off was 0.05 for retention and elimination of variables in the model. All statistical analyses were performed using the Statistical Package for the Social Sciences (SPSS) for Windows software package, version 22.0 (SPSS Inc., Chicago, IL, USA). Two-tailed *P*-values of < 0.05 were considered statistically significant.

## Additional Information

**How to cite this article:** Duan, D. *et al*. Empty-nest-related psychological distress is associated with progression of brain white matter lesions and cognitive impairment in the elderly. *Sci. Rep.* **7**, 43816; doi: 10.1038/srep43816 (2017).

**Publisher's note:** Springer Nature remains neutral with regard to jurisdictional claims in published maps and institutional affiliations.

## Supplementary Material

Supplementary Table

## Figures and Tables

**Table 1:** Characteristics and demographics of participants at baseline.

	Control group (*n* = 83)	Couples group (*n* = 70)	Single group (*n* = 66)	*P* value
Age, years	69.69 ± 5.75	70.46 ± 4.81	69.41 ± 5.83	0.140
Sex, F/M	39/44	35/35	37/29	0.541
Drinker, *n* (%)	30 (36.14)	19 (27.14)	14 (21.21)	0.127
Smoker, *n* (%)	9 (10.84)	4 (5.71)	9 (13.64)	0.293
Education, years	9.70 ± 4.29	9.41 ± 3.76	7.82 ± 4.48*	0.018
Hypertension history, *n* (%)	56 (67.47)	56 (80.00)	61 (92.42)*^,†^	0.001
Antihypertension, *n* (%)	44 (78.57)	51 (91.07)	54 (88.52)	0.096
Diabetes history, *n* (%)	11 (13.25)	7 (10.00)	6 (9.09)	0.687
Anti-diabetes, *n* (%)	11 (100.00)	7 (100.00)	6 (100.00)	1.000
Body mass index, kg/m^2^	24.91 ± 3.23	24.79 ± 3.43	25.06 ± 3.64	0.898
Systolic blood pressure, mm Hg	137.58 ± 14.41	145.37 ± 16.87*	154.89 ± 13.65*^,†^	<0.001
Diastolic blood pressure, mm Hg	70.04 ± 10.07	73.64 ± 10.37	71.68 ± 11.19	0.111
Total cholesterol, mmol/L	4.63 ± 0.87	4.72 ± 0.78	4.91 ± 0.69	0.102
Triglycerides, mmol/L	1.56 ± 0.55	1.57 ± 0.64	1.63 ± 0.57	0.737
High-density lipoprotein cholesterol, mmol/L	1.24 ± 0.31	1.22 ± 0.29	1.22 ± 0.31	0.882
Low-density lipoprotein cholesterol, mmol/L	2.72 ± 0.65	2.81 ± 0.56	2.95 ± 0.52	0.060
Fasting plasma glucose, mmol/L	5.43 ± 0.70	5.53 ± 0.67	5.48 ± 0.67	0.689

Results are presented as the mean ± standard deviation. *P < 0.05, compared to control group; ^†^*P* < 0.05, compared to couples group.

**Table 2:** Baseline variables of psychological outcome, global cognitive function, and brain MRI.

	Control group (*n* = 83)	Couples group (*n* = 70)	Single group (*n* = 66)	*P* value
Psychological outcome				
UCLA-LS score, points	29.64 ± 6.53	36.13 ± 7.01*	47.20 ± 7.31*^,†^	<0.001
GDS score, points	4.45 ± 2.45	5.77 ± 2.68*	8.50 ± 2.72*^,†^	<0.001
Global cognitive function				
MMSE score, points	28.00 (26.00, 29.00)	27.00 (26.75, 28.00)	27.00 (26.00, 28.00)	0.087
MoCA score, points	27.00 (26.00, 28.00)	26.00 (26.00, 28.00)	26.00 (25.00, 27.00)	0.063
Brain MRI				
PWMH, mL	4.66 ± 2.91	5.06 ± 2.81	4.93 ± 2.65	0.675
DWMH, mL	1.66 ± 0.86	1.64 ± 0.94	1.53 ± 0.87	0.475
Total WMH, mL	6.32 ± 3.33	6.70 ± 3.22	6.46 ± 3.05	0.766

Results are presented as the mea ersity of California at Los Angeles Loneliness Scale; GDS, Geriatric Depression Scale; MMSE, Mini-Mental State Examination; MoCA, Montreal Cognitive Assessment; PWMH, periventricular white matter hyperintensities; DWMH, deep white matter hyperintensities; WMH, white matter hyperintensities. *P < 0.05, compared to control group; ^†^*P* < 0.05, compared to couples group.

**Table 3:** Correlation of psychological outcome with global cognitive function and brain WMH in all participants.

	Change of MMSE		Change of MoCA		Change of PWMH		Change of DWMH		Change of total WMH	
	Correlation coefficient	*P* value	Correlation coefficient	*P* value	Correlation coefficient	*P* value	Correlation coefficient	*P* value	Correlation coefficient	*P* value
Differences between baseline and the final follow-up										
UCLA-LS score, points	−0.487*	<0.001	−0.531	<0.001	0.259	<0.001	0.248	<0.001	0.289	<0.001
GDS score, points	−0.389*	<0.001	0.434	<0.001	0.249	<0.001	0.283	<0.001	0.267	<0.001
Change percentages over a 5-year follow-up period										
UCLA-LS score, points	−0.495	<0.001	−0.536	<0.001	0.599	<0.001	0.467	<0.001	0.612	<0.001
GDS score, points	−0.393	<0.001	−0.439	<0.001	0.449	<0.001	0.401	<0.001	0.484	<0.001

*Spearman correlation coefficients. Unit of difference of variables between baseline and the final follow-up: change of MMSE, point; change of MoCA, point; change of PWMH, mL; change of DWMH, mL; change of WMH, mL. Unit of change percentage of variables between baseline and final follow-up, %. UCLA-LS, University of California at Los Angeles Loneliness Scale; GDS, Geriatric Depression Scale; MMSE, Mini-Mental State Examination; MoCA, Montreal Cognitive Assessment; PWMH, periventricular white matter hyperintensities; DWMH, deep white matter hyperintensities; WMH, white matter hyperintensities.

**Table 4:** Associations of psychological outcome with global cognitive function and brain WMH in all participants after adjusting for confounders.

	Change of MMSE*		Change of MoCA*		Change of PWMH^†^		Change of DWMH^†^		Change of total WMH^†^	
	Beta coefficient (95% C.I.)	*P* value	Beta coefficient (95% C.I.)	*P* value	Beta coefficient (95% C.I.)	*P* value	Beta coefficient (95% C.I.)	*P* value	Beta coefficient (95% C.I.)	*P* value
Differences between baseline and the final follow-up										
UCLA-LS score, point	−0.060 (−0.080, −0.040)	<0.001	−0.050 (−0.072, −0.029)	<0.001	0.012 (0.001, 0.022)	0.027	0.002 (0.001, 0.003)	0.001	0.013 (0.001, 0.024)	0.027
GDS score, point	−0.052 (−0.093, −0.011)	0.010	−0.066 (−0.112, 0.020)	0.037	0.030 (0.009, 0.051)	0.008	0.015 (0.008, 0.022)	<0.001	0.039 (0.004, 0.074)	0.028
Change percentages over a 5-year follow-up period										
UCLA-LS score, points	−0.218 (−0.291, −0.146)	<0.001	−0.184 (−0.266, −0.103)	<0.001	0.347 (0.281, 0.413)	<0.001	0.190 (0.099, 0.282)	<0.001	0.271 (0.199, 0.344)	<0.001
GDS score, points	−0.214 (−0.361, −0.67)	<0.001	−0.255 (−0.414, −0.096)	0.017	0.179 (0.096, 0.262)	<0.001	0.270 (0.116, 0.424)	<0.001	0.214 (0.075, 0.353)	<0.001

Independent variables include age, sex, smoking (yes or no), alcohol consumption (yes or no), education, body mass index, history of hypertension (yes or no), use of antihypertensive agents (yes or no), history of diabetes mellitus (yes or no), use of hypoglycemic agents (yes or no), blood pressure, fasting blood lipid and glucose levels, UCLA-LS, and GDS. *Independent variables also include baseline total WMH. ^†^Independent variables also include baseline MMSE score. Unit of difference of variables between baseline and the final follow-up: change of MMSE, point; change of MoCA, point; change of PWMH, mL; change of DWMH, mL; change of WMH, mL. Unit of change percentage of variables between baseline and final follow-up, %. UCLA-LS, University of California at Los Angeles Loneliness Scale; GDS, Geriatric Depression Scale; MMSE, Mini-Mental State Examination; MoCA, Montreal Cognitive Assessment; PWMH, periventricular white matter hyperintensities; DWMH, deep white matter hyperintensities; WMH, white matter hyperintensities.

## References

[b1] Liang Y. & Wu W. Exploratory analysis of health-related quality of life among the empty-nest elderly in rural China: An empirical study in three economically developed cities in eastern China. Health Qual. Life Out 12, 59 (2014). 10.1186/1477-7525-12-59 PMC4016644 24766880

[b2] Zhou C. . Non-use of health care service among empty-nest elderly in Shandong, China: a cross-sectional study. BMC Health Serv. Res. 15, 294 (2015). 26219288 10.1186/s12913-015-0974-1 PMC4517420

[b3] Wu Z. Q. . Correlation between loneliness and social relationship among empty nest elderly in Anhui rural area. Aging Ment. Health. 4, 108–112 (2010). 10.1080/13607860903228796 20155527

[b4] Gratton B. & Gutmann M. P. Emptying the nest: older men in the United States, 1880–2000. Popul. Dev. Rev. 36, 331–356 (2010). 20734555 10.1111/j.1728-4457.2010.00332.x

[b5] Liu L. J. & Guo Q. Life satisfaction in a sample of empty-nest elderly: a survey in the rural area of a mountainous county in China. Qual. Life Res. 17, 823–830 (2008). 18595006 10.1007/s11136-008-9370-1

[b6] Yang T. . Catastrophic health expenditure: a comparative analysis of empty-nest and non-empty-nest households with seniors in Shandong, China. BMJ Open. 6, e010992 (2016). 10.1136/bmjopen-2015-010992 PMC4947795 27381206

[b7] Eloranta S., Arve S., Isoaho H., Lehtonen A. & Viitanen M. Loneliness of older people aged 70: a comparison of two Finnish cohorts born 20 years apart. Arch. Gerontol. Geriatr. 61, 254–260 (2015). 26143336 10.1016/j.archger.2015.06.004

[b8] Liu L. J. & Guo Q. Loneliness and health-related quality of life for the empty nest elderly in the rural area of a mountainous county in China. Qual. Life Res. 16, 1275–1280 (2007). 17703375 10.1007/s11136-007-9250-0

[b9] Copeland J. R. . Depression among older people in Europe: the EURODEP studies. World Psychiat. 3, 45–49 (2004). PMC1414664 16633454

[b10] Villarreal A. E., Grajales S., Lopez L., Britton G. B. & Initiative P. A. Cognitive impairment, depression, and cooccurrence of both among the elderly in Panama: differential associations with multimorbidity and functional limitations. Biomed. Res. Int. 2015, 718701 (2015). 26798641 10.1155/2015/718701 PMC4698525

[b11] Momtaz Y. A. . Loneliness as a risk factor for hypertension in later life. J. Aging Health. 24, 696–710 (2012). 22422758 10.1177/0898264311431305

[b12] Thurston R. C. & Kubzansky L. D. Women, loneliness and incident coronary heart disease. Psychosom. Med. 71, 836–842 (2009). 19661189 10.1097/PSY.0b013e3181b40efc PMC2851545

[b13] Yoon K. H. . The relationship between serum endocan levels and depression in Alzheimer’s deisease. Dis. Markers. 2016, 8254675 (2016). 26924874 10.1155/2016/8254675 PMC4746338

[b14] Whisman M. A. Loneliness and the metabolic syndrome in a population-based sample of middle-aged and older adults. Health Psychol. 29, 550–554 (2010). 20836610 10.1037/a0020760

[b15] Duane F., Brasher K. & Koch S. Living alone with dementia. Dementia. 12, 123–136 (2013). 24336667 10.1177/1471301211420331

[b16] Dregan A., Stewart R. & Gulliford M. C. Cardiovascular risk factors and cognitive decline in adults aged 50 and over: a population-based cohort study. Age Ageing. 42, 338–345 (2013). 23179255 10.1093/ageing/afs166

[b17] Johnson K. C. . A prospective study of the effect of hypertension and baseline blood pressure on cognitive decline and dementia in postmenopausal women: the Women’s Health Initiative Memory Study. J. Am. Geriatr. Soc. 56, 1449–1458 (2008). 18637980 10.1111/j.1532-5415.2008.01806.x

[b18] Okusaga O. . Smoking, hypercholesterolaemia and hypertension as risk factors for cognitive impairment in older adults. Age Ageing. 42, 306–311 (2013). 23302603 10.1093/ageing/afs193

[b19] Nguyen M. Nurse’s assessment of caregiver burden. Medsurg. Nurs. 18, 147–151 (2009). 19591360

[b20] Silbert L. C. . Trajectory of white matter hyperintensity burden preceding mild cognitive impairment. Neurology. 79, 741–747 (2012). 22843262 10.1212/WNL.0b013e3182661f2b PMC3421153

[b21] Liu Z. . Low carotid artery wall shear stress is independently associated with brain white-matter hyperintensities and cognitive impairment in older patient. Aterosclerosis. 247, 78–86 (2016). 10.1016/j.atherosclerosis.2016.02.003 26868512

[b22] Liu Z. . Excessive variability in systolic blood pressure that is self-measured at home exacerbates the progression of brain white matter lesions and cognitive impairment in the oldest old. Hypertens. Res. 39, 245–253 (2016). 26631851 10.1038/hr.2015.135

[b23] Peng J. . Excessive lowering of blood pressure is not beneficial for progression of brain white matter hyperintensive and cognitive impairment in elderly hypertensive patients: 4-year follow-up study. J. Am. Med. Dir. Assoc. 15, 904–910 (2014). 25239015 10.1016/j.jamda.2014.07.005

[b24] O’Luanaigh C. . Loneliness and cognition in older people: the Dublin Healthy Ageing study. Aging Ment. Health. 16, 347–352 (2012). 22129350 10.1080/13607863.2011.628977

[b25] Johansson L. . Midlife psychological distress associated with late-life brain atrophy and white matter lesions: a 32-year population study of women. Psychosom. Med. 74, 120–125 (2012). 22286853 10.1097/PSY.0b013e318246eb10

[b26] Taylor W. D. . White matter hyperintensity progression and late-life depression outcomes. Arch. Gen. Psychiatry. 60, 1090–1096 (2003). 14609884 10.1001/archpsyc.60.11.1090

[b27] Fujishima M. . Mild cognitive impairment, poor episodic memory, and late-life depression are associated with cerebral cortical thinning and increased white matter hyperintensities. Front. Aging Neurosci. 6, 306 (2014). 25426066 10.3389/fnagi.2014.00306 PMC4224123

[b28] Nakagawa S. . White matter structures associated with loneliness in young adults. Sci. Rep. 5, 17001 (2015). 26585372 10.1038/srep17001 PMC4653806

[b29] Kuiper J. S. . Social relationships and risk of dementia: A systematic review and meta-analysis of longitudinal cohort studies. Ageing Res. Rev. 22, 39–57 (2015). 25956016 10.1016/j.arr.2015.04.006

[b30] Santini Z. I., Koyanagi A., Tyrovolas S., Mason C. & Haro J. M. The association between social relationships and depression: a systematic review. J. Affect. Disord. 175, 53–65 (2014). 25594512 10.1016/j.jad.2014.12.049

[b31] Sousa M., Pereira A. & Costa R. Subjective memory complaint and depressive symptoms among older adults in Portugal. Curr. Gerontol. Geriatr. Res. 2015, 296581 (2015). 26880907 10.1155/2015/296581 PMC4735984

[b32] Pan C. W. . Cognitive dysfunction and health-related quality of life among older Chinese. Sci. Rep. 5, 17301 (2015). 26601612 10.1038/srep17301 PMC4658548

[b33] Stern Y. Cognitive reserve in ageing and Alzheimer’s disease. Lancet Neurol. 11, 1006–1012 (2012). 23079557 10.1016/S1474-4422(12)70191-6 PMC3507991

[b34] Fratiglioni L., Paillar-Borg S. & Winblad B. An active and socially integrated lifestyle in late life might protect against dementia. Lancet Neruol. 3, 343–353 (2004). 10.1016/S1474-4422(04)00767-7 15157849

[b35] Scarmeas N. & Stern Y. Cognitive reserve and lifestyle. J. Clin. Exp. Neuropsychol. 25, 625–633 (2003). 12815500 10.1076/jcen.25.5.625.14576 PMC3024591

[b36] Wilson R. S. . Proneness to psychological distress is associated with risk of Alzheimer’s disease. Neurology. 61, 1479–1485 (2003). 14663028 10.1212/01.wnl.0000096167.56734.59

[b37] Brickman A. M., Muraskin J. & Zimmerman M. E. Structural neuroimaging in Alzheimer’s disease: do white matter hyperintensities matter? Dialogues. Clin. Neurosci. 11, 181–190 (2009). 19585953 10.31887/DCNS.2009.11.2/ambrickman PMC2864151

[b38] van den Heuvel D. M. . Increase in periventricular white matter hyperintensities parallels decline in mental processing speed in a non-demented elderly population. J. Neurol. Neuosurg. Psychialtry. 77, 149–153 (2006). 10.1136/jnnp.2005.070193 PMC2077562 16421114

[b39] Sachdev P. & Wen W. Should we distinguish between periventricular and deep white matter hyperintensities? Stroke. 36, 2342–2344 (2005). 16239634 10.1161/01.STR.0000185694.52347.6e

[b40] Sachdev P. S. . White matter hyperintensities are under strong genetic influence. Stroke. 47, 1422–1428 (2016). 27165950 10.1161/STROKEAHA.116.012532

[b41] Smith C. D. . Peripheral (deep) but not periventicular MRI white matter hyperintensities are increased in clinical vascular dementia compared to Alzheimer’s desease. Brain Behav. 6, e00438 (2016). 26925303 10.1002/brb3.438 PMC4754499

[b42] Kaszniak A. W., Garron D. C., Fox J. H., Bergen D. & huckman M. Cerebral atrophy, EEG, slowing age, education, and cognitive functioning in suspected dementia. Neurology. 29, 1273–1279 (1979). 573407 10.1212/wnl.29.9_part_1.1273

[b43] Tai S. Y., Wang L. C. & Yang Y. H. Effect of music intervention on the cognitive and depression status of senior apartment residents in Taiwan. Neuropsychiatr. Dis. Treat. 11, 1446–1454 (2015). 10.2147/NDT.S82572 PMC4472066 26109859

[b44] Sheikh J. I. . Proposed factor structure of the Geriatric Depression Scale. Int. Psychogeriatr. 3, 23–28 (1991). 1863703 10.1017/s1041610291000480

[b45] O’Hare K. . Links between depressive symptoms and unmet health and social care needs among older prisoners. Age Ageing. 45, 158–163 (2016). 26764402 10.1093/ageing/afv171 PMC4711658

[b46] Wang C. . Association between exposure to the Chinese famine in different stages of early life and decline in cognitive functioning in adulthood. Front Behav. Neurosci. 10, 146 (2016). 27471454 10.3389/fnbeh.2016.00146 PMC4943926

[b47] Yaneva-Sirakova T., Tarnovska-Kadreva R. & Traykov L. The role of suboptimal home-measured blood pressure control for cognitive decline. Dement. Geriatr. Cogn. Disord. Extra. 2, 112–119 (2012). 10.1159/000337502 PMC3347878 22590472

[b48] Tu Q. Y. . Reliability, validity, and optimal cutoff score of the Montreal Cognitive Assessment (Changsha Version) in ischemic cerebrovascular disease patients of Hunan province, China. Dement. Geriatr. Cogn. Dis. Extra. 3, 25–36 (2013). 23637698 10.1159/000346845 PMC3617974

[b49] Fischl B. . Whole brain segmentation: automated labeling of neuroanatomical structures in the human brain. Neuron. 33, 341–355 (2002). 11832223 10.1016/s0896-6273(02)00569-x

