# Supplementary material for: Empty-nest-related psychological distress is associated with progression of brain white matter lesions and cognitive impairment in the elderly
Source: Sci Rep. 2017 Mar 3;7:43816. doi: 10.1038/srep43816 (PMC5335556; doi:10.1038/srep43816)
Supplement: Supplementary Table [file srep43816-s1.doc]

**Title of Manuscript:** Empty-nest related psychological disorders exacerbate the progression of brain white matter lesions and cognitive impairment in elderly

**Author List:** Dandan Duan, Yuanli Dong, Hua Zhang, Yingxin Zhao, Yutao Diao, Yi Cui, Juan Wang, Qiang Chai, Zhendong Liu

|  | Change of MMSE | | |  | Change of MoCA | | |  | Change of PWMH | | |  | Change of DWMH | | |  | Change of total WMH | | |
| --- | --- | --- | --- | --- | --- | --- | --- | --- | --- | --- | --- | --- | --- | --- | --- | --- | --- | --- | --- |
|  | Beta coefficient (95% C.I.) | *P* value | Adjusted R Square |  | Beta coefficient (95% C.I.) | *P* value | Adjusted R Square |  | Beta coefficient (95% C.I.) | *P* value | Adjusted R Square |  | Beta coefficient (95% C.I.) | *P* value | Adjusted R Square |  | Beta coefficient (95% C.I.) | *P* value | Adjusted R Square |
| a. Differences between baseline and the final follow-up | | | | | | | | | | | | | | | | | | | |
| UCLA-LS score, point | -0.060  (-0.080, -0.040) | < 0.001 | 0.344 |  | -0.050  (-0.072, -0.029) | < 0.001 | 0.363 |  | 0.012  (0.001, 0.022) | 0.027 | 0.113 |  | 0.002  (0.001, 0.003) | 0.001 | 0.107 |  | 0.013  (0.001, 0.024) | 0.027 | 0.135 |
| GDS score, point | -0.052  (-0.093, -0.011) | 0.010 |  |  | -0.066  (-0.112,-0.020) | 0.037 |  |  | 0.030  (0.009, 0.051) | 0.008 |  |  | 0.015  (0.008, 0.022) | < 0.001 |  |  | 0.039  (0.004, 0.074) | 0.028 |  |
| Education, years | 0.117  (0.073, 0.161) | < 0.001 |  |  | 0.086  (0.048, 0.124) | < 0.001 |  |  | -- | -- |  |  | -- | -- |  |  | -- | -- |  |
| Low-density lipoprotein cholesterol, mmol/L | -0.339 (-0.646, -0.032) | 0.030 |  |  | -- | -- |  |  | -- | -- |  |  | -- | -- |  |  | -- | -- |  |
| Baseline total WMH, mL | -0.066 (-0.122, -0.010) | 0.022 |  |  |  |  |  |  |  |  |  |  |  |  |  |  | -- | -- |  |
| Total cholesterol, mmol/L | -- | -- |  |  | -- | -- |  |  | -- | -- |  |  | 0.077  (0.002, 0.152) | 0.045 |  |  |  |  |  |
| Fasting plasma glucose, mmol/L | -- | -- |  |  | -- | -- |  |  | -- | -- |  |  | 0.042  (0.010, 0.074) | 0.010 |  |  | 0.129  (0.004, 0.254) | 0.044 |  |
| Systolic blood pressure, mm Hg | -- | -- |  |  | -- | -- |  |  | 0.007 (0.000, 0.014) | 0.033 |  |  | -- | -- |  |  | -- | -- |  |
| Hypertension history | -- | -- |  |  | -- | -- |  |  | 0.344  (0.097, 0.591) | 0.007 |  |  | -- | -- |  |  | 0.340  (0.065, 0.616) | 0.016 |  |
| Body mass index, kg/m^2^ | -- | -- |  |  | -- | -- |  |  | -- | -- |  |  | 0.006  (0.000, 0.012) | 0.048 |  |  | -- | -- |  |
| Antihypertension, yes/no | -- | -- |  |  | 0.597  (0.095, 1.100) | 0.020 |  |  | -0.250  (-0.494, -0.006) | 0.044 |  |  | -0.093  (-0.161, -0.026) | 0.007 |  |  | -0.330  (-0.602, -0.058) | 0.018 |  |
| b. Change in percentages between baseline and the final follow-up | | | | | | | | | | | | | | | | | | | |
| UCLA-LS score, point | -0.218  (-0.291, -0.146) | < 0.001 | 0.361 |  | -0.184  (-0.266, -0.103) | < 0.001 | 0.378 |  | 0.347  (0.281, 0.413) | < 0.001 | 0.382 |  | 0.190  (0.099, 0.282) | < 0.001 | 0.261 |  | 0.271  (0.199, 0.344) | < 0.001 | 0.406 |
| GDS score, point | -0.214  (-0.361, -0.67) | < 0.001 |  |  | -0.255  (-0.414, -0.096) | 0.017 |  |  | 0.179  (0.096, 0.262) | < 0.001 |  |  | 0.270  (0.116, 0.424) | < 0.001 |  |  | 0.214  (0.075, 0.353) | < 0.001 |  |
| Education, years | 0.443  (0.283, 0.603) | < 0.001 |  |  | 0.354  (0.209, 0.498) | < 0.001 |  |  | -- | -- |  |  | -- | -- |  |  | -- | -- |  |
| Low-density lipoprotein cholesterol, mmol/L | -1.180  (-2.302, -0.058) | 0.039 |  |  | -- | -- |  |  | -- | -- |  |  | -- | -- |  |  | -- | -- |  |
| Baseline total WMH, mL | -0.258 (-0.464, -0.052) | 0.014 |  |  |  |  |  |  |  |  |  |  |  |  |  |  |  |  |  |
| Antihypertension, yes/no | -- | -- |  |  | 2.435  (0.515, 4.355) | 0.013 |  |  | -1.916  (-3.307, -0.526) | 0.007 |  |  | -- | -- |  |  | -1.744  (-2.961, -0.528) | 0.005 |  |
| Body mass index, kg/m^2^ | -- | -- |  |  | -- | -- |  |  | -- |  |  |  | 0.218  (0.011, 0.425) | 0.039 |  |  | -- | -- |  |
| Fasting plasma glucose, mmol/L | -- | -- |  |  | -- | -- |  |  | -- | -- |  |  | 0.947  (0.074, 1.820) | 0.033 |  |  | -- | -- |  |

**Supplement Table 1 Factors possibly associated with global cognitive function and brain WMH in all participants using multiple linear backward stepwise regression analysis.** Independent variables include age, sex, smoking (yes or no), alcohol consumption (yes or no), education, body mass index, history of hypertension (yes or no), use of antihypertensive agents (yes or no), history of diabetes mellitus (yes or no), use of hypoglycemic agents (yes or no), blood pressure, fasting blood lipid and glucose levels, UCLA-LS, and GDS. * Independent variables also include baseline total WMH. ^†^ Independent variables also include baseline MMSE score. Unit of difference of variables between baseline and the final follow-up: change of MMSE, point; change of MoCA, point; change of PWMH, mL; change of DWMH, mL; change of WMH, mL. Unit of change percentage of variables between baseline and final follow-up, %. UCLA-LS, University of California at Los Angeles Loneliness Scale; GDS, Geriatric Depression Scale; MMSE, Mini-Mental State Examination; MoCA, Montreal Cognitive Assessment; PWMH, periventricular white matter hyperintensities; DWMH, deep white matter hyperintensities; WMH, white matter hyperintensities.
